# Evolution of the RAG1-RAG2 locus: both proteins came from the same transposon

**DOI:** 10.1186/s13062-015-0055-8

**Published:** 2015-04-28

**Authors:** Vladimir V Kapitonov, Eugene V Koonin

**Affiliations:** National Center for Biotechnology Information, National Library of Medicine, National Institutes of Health, Bethesda, MD USA

**Keywords:** Molecular evolution, genetics, immune system, V(D)J recombination, RAG1 and RAG2 proteins, *Transib* DNA transposons, Transib transposase

## Abstract

**Abstract:**

The RAG1 and RAG2 proteins are essential subunits of the V(D)J recombinase that is required for the generation of the enormous variability of antibodies and T-cell receptors in jawed vertebrates. It was demonstrated previously that the 600-aa catalytic core of RAG1 evolved from the transposase of the *Transib* superfamily transposons. However, although homologs of RAG1 and RAG2 genes are adjacent in the purple sea urchin genome, a transposon encoding both proteins so far has not been reported. Here we describe such transposons in the genomes of green sea urchin, a starfish and an oyster. Comparison of the domain architectures of the RAG1 homologs in these transposons, denoted *TransibSU*, and other *Transib* superfamily transposases provides for reconstruction of the structure of the hypothetical *TransibVDJ* transposon that gave rise to the VDJ recombinases at the onset of vertebrate evolution some 500 million years ago.

**Reviewers:**

This article was reviewed by Mart Krupovic and I. King Jordan.

**Electronic supplementary material:**

The online version of this article (doi:10.1186/s13062-015-0055-8) contains supplementary material, which is available to authorized users.

## Findings

RAG1 and RAG2 proteins constitute the enzymatic core of the V(D)J recombination machinery in jawed vertebrates [1-4]. The RAG1-RAG2 complex catalyzes random assembly of Variable, Diverse, and Joining gene segments that are present in the genome in numerous copies and, together with hypermutation, generate the enormous variety of the assembled antibodies and antigen receptors [5-7]. We have shown previously that the 600-aa catalytic core of RAG1 and VDJ recombination signal sequences (RSS) has evolved from the transposase and terminal inverted repeats (TIRs) of a *Transib* superfamily transposon, respectively, and this event has been mapped to the common ancestor of jawed vertebrates that lived about 500 million years ago (MYA) [8]. The RAG2 protein adopts a six-bladed beta-propeller structure and also contains a PHD finger domain; this protein is involved in binding the RSS [9-11]. The recent breakthrough report of the crystal structure of the RAG1-RAG2 heterotetramer supports the architectural similarity of the V(D)J recombinase with transposases and provides for a detailed model of the interaction of the complex with the RSS [12]. So far RAG2 has not been detected in transposable elements.

**Figure 1:**
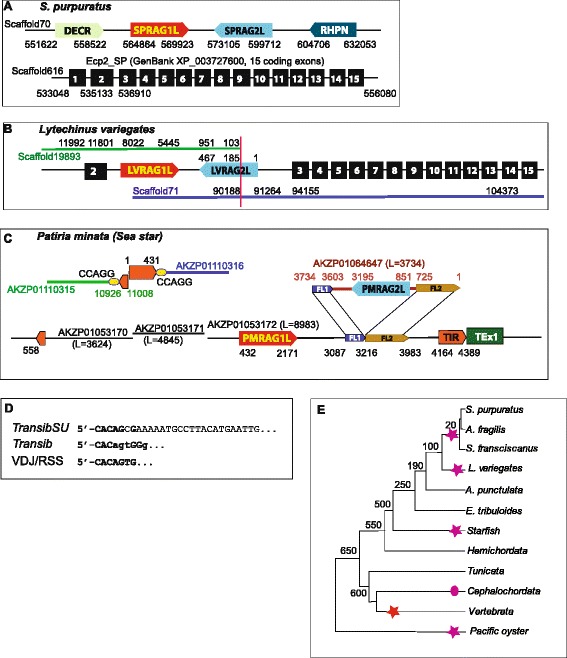
*TransibSU* transposons in sea urchins and starfish. **A**: The RAG1-RAG2-like locus in the purple sea urchin genome. DECR (GenBank: XP_793296) and RHPN (GenBank: XP_785878) are neighbor genes flanking the RAG1-RAG2-like locus. Numbers indicate positions of SPRAG1L, SPRAG2L and their flanking genes in Scaffold70 (GenBank: NW_003577031). Scaffold616 (GenBank: NW_003577577) encodes the elongator complex protein 2 gene (Ecp2, conserved in all metazoans). Numbers indicate the beginning and the end of the coding exons (black rectangles) and position of the second intron (between the exons 2 and 3). **B**: In the green sea urchin *L. variegates* genome, a unit coding for both LVRAG1L and LVRAG2L is inserted into the second intron of Ecp2, this gene is orthologous to the purple sea urchin Ecp2. Both genomes contain a single copy of the Ecp2 gene. The green sea urchin LVRAG1L/LVRAG2L unit is assembled from two scaffolds. Scaffold positions corresponding to loci coding for Ecp2, LVRAG1L and LVRAG2L are shown above and the green (Scaffold19893) and black (Scaffold71) lines, respectively. The N-terminal part of LVRAG1L is lost (1-272-aa of SPRAG1), the core and C-terminal part is almost intact (it corresponds to pos. 437–978 of SPRAG1; disrupted by one stop codon). The vertical red line indicates the boundary between the LVRAG2L parts (aa positions 1–184 and 185–467), encoded by the two scaffolds due to incomplete assembly. **C**: The *TransibSU* transposons in the Bat star genome (*Patiria minata*, sea star or starfish). One copy of the *TransibSU-1_PM* transposon can be assembled from three contigs [GenBank: AKZP01053170-AKZP01054172]. The RAG2L protein in this copy was lost due to deletion of a region between the FL1 and FL2 parts (dark blue and brown arrows). The 94% identical copies of FL1 and FL2 constitute the termini of a 3734-bp contig [GenBank: AKZP01064647]. The central part of this contig encodes the PMRAG2L protein. This contig represents a part of the second copy of the *TransibSU-1_PM* transposon. A copy of the second *TransibSU-2_PM* transposon is present at the 3’ and 5’ termini of the AKZP01110315 (green line) and AKZP01110316 (blue line) contigs, which are assembled into one scaffold. This transposon is flanked by the CCAGG target site duplication (yellow ovals). Due to the sequencing problems, the internal part of this transposon is not complete. **D**: Termini of *TransibSU*, *Transibs* and V(D)J recombination signal sequences are shown. **E**: Commonly accepted phylogeny of species colonized by the *TransibSU* and *TransibVDJ* transposons. Magenta stars denote the presence of *TransibSU* transposons and the red stars denote the RAG1-RAG2 locus. The magenta oval indicates an unknown RAG1-RAG2-enocding transposon reported recently as a polymorphic insertion in a lancelet genome [24].

All known *Transib* transposons encode only one protein, the Transib transposase. The purple Sea urchin *Strongylocentrotus purpuratus* genome encompasses a RAG1-RAG2-like locus (Figure 1A) in which the genes for both proteins located in close proximity, in the head-to-head orientation; however, this locus lacks TIRs and thus does not show typical features of a transposon [13]. The vertebrate RAG1 proteins show a substantially greater sequence similarity to the sea urchin RAG1-like protein (SPRAG1L) than to the known Transib transposases. Accordingly, it has been suggested that the ancestral RAG1-RAG2 locus existed already in the common ancestor of the deuterostomes >600 MYA and was subsequently lost in many lineages including jawless vertebrates, *Cephalochordata* and *Tunicata* [13].

Here we show that both RAG1 and RAG2 subunits of the VDJ recombinase evolved from two proteins encoded in a single transposon which we accordingly denote *TransibVDJ*.

### The RAG1L-RAG2L locus is inserted into an intron of the elongator complex protein 2 gene in the green sea urchin genome

In the recently sequenced genome of the green sea urchin (*Lytechinus variegatus*), a protein with a high similarity to SPRAG1L (50% identity) is encoded in the Scaffold198 that additionally encodes a protein similar to the N-truncated SPRAG2 (Figure 1B). Analogous to RAG1-RAG2 and SPRAG1L-SPRAG2L, these two proteins are encoded in a head-to-head orientation and close to each other (a 4493-bp spacer). The remaining 184-aa N-terminal portion of the SPRAG2L-like protein is encoded by Scaffold71. Apparently, Scaffold198, in the reverse orientation, and Scaffold71 should be assembled into a single locus encoding the LVRAG1L and LVRAG2L proteins (Figure 1B). This approximately 10 kb locus is inserted into the second intron (between exons 2 and 3) of the gene for the elongator complex protein 2 (Ecp2) which is conserved in all metazoans (Figure 1B).

Both sea urchin genomes encompass a single copy of the Ecp2 gene. However, the RAG1L and RAG2L proteins are encoded only in a green sea urchin Ecp2 gene intron. The green sea urchin LVRAG1L gene appears to be non-functional given the loss of ~200-aa N-terminal portion, interruption of the coding sequence by premature stop codons and frameshifts, which is typical of most inactive transposons fossilized in the genome [8]. The intact-like LVRAG2L is encoded by 3 exons similar to the SPRAG2L gene in the purple sea urchin (see Additional file 1). The insertion of the LVRAG1L and LVRAG2L into an intron is compatible with the two genes coming from the same transposon although TIRs that would mark the ends of such a hypothetical transposon were not identified. The preservation of the gene organization along with the absence of the RAG1-RAG2 locus in the corresponding intron of *S. purpuratus* imply that the insertion of the transposon in the green sea urchin occurred after its split from the purple sea urchin some 50 MYA [14]. Furthermore, none of the genes that flank SPRAG1L and SPRAG2L in the purple sea urchin are associated with the LVRAG1-LVRAG2 locus in the green sea urchin (Figure 1A). Thus, SPRAG1L-SPRAG2L and LVRAG1L-LVRAG2L appear to derive from two related but distinct transposons that most likely independently inserted into the purple and green sea urchin genomes a few million years ago. These two hypothetical transposons represent a new group within the *Transib* superfamily. The distinctive feature of this group, hereinafter denoted *TransibSU* (after Sea Urchin), is the presence of both RAG1 and RAG2 genes.

For reasons that remain to be understood, autonomous *Transib* transposons are typically present in animal genomes in only one or at most a few copies [8]. Therefore, it is not surprising that the termini of the green and purple sea urchin *TransibSU* transposons that apparently inserted millions of years ago into the Ecp2 intron and in the spacer between the DECR and RHPN genes, respectively, and were then fossilized, are not detectable.

### Identification of a TransibSU transposon in the Bat star genome

In the assembly of the recently sequenced Bat star genome, we identified a contig encoding a protein (PMRAG1L) that is 50% identical to SPRAG1L (Figure 1C). This contig could be linked to another short contig that encodes a RAG2 homolog (PMRAG2L) (Figure 1C). Indeed, two juxtaposed regions in the PMRAG1L-encoding contig were 94% identical to the terminal regions in the PMRAG2 contig (Figure 1C). The most parsimonious explanation for this link is the existence of two >90% identical copies of an autonomous *TransibSU* transposon encoding PMRAG1L and PMRAG2L in the head-to-head orientation. In one of the copies, the PMRAG2L-coding region apparently was deleted. The second copy is not sequenced completely and thus might also encode the PMRAG1L and possibly contain the transposon termini. Both PMRAG1L and PMRAG2L genes are composed of three exons (Additional file 2).

In an attempt to identify the termini of the transposon, we analyzed the DNA sequences flanking the PMRAG1L gene by using these sequences as queries in a BLASTN-based Censor search against all contigs representing >85% of the ~800-mb *P. minata* genome. This search resulted in the identification of two groups of sequences, >140 bp and >200 bp in length, that are repeated only 10 and 7 times in the *P. minata* genome, and have perfectly defined 5’- and 3’-boundaries, respectively (Figure 1C and Additional file 3). These two boundaries appear to represent the 5’- and 3’-ends of the *TransibSU-1_PM* transposon, respectively. Analogous to the known *Transibs*, these ends contain 13-bp identical TIRs (Figure 1C and Additional file 3). In one case, the termini of *TransibSU-1_PM* were identified in adjacent contigs and were flanked by 5 bp target site duplicates that are typical of *Transibs* (Figure 1C). Notably, the 7 bp termini of *TransibSU-1_PM* are closely similar to the termini of known *Transibs* and to the RSS at the vertebrate V(D)J junctions (Figure 1D) [8]. Thus, analysis of the starfish genome sequence presents the first direct evidence of the existence of transposons that encode both RAG1 and RAG2.

In addition to the Echinoderms (sea urchins and starfish), a damaged coding core of a *TransibSU* transposon was identified also in the genome of the mollusk *Crassostrea gigas* (Pacific oyster) (Additional file 4).

**Figure 2:**
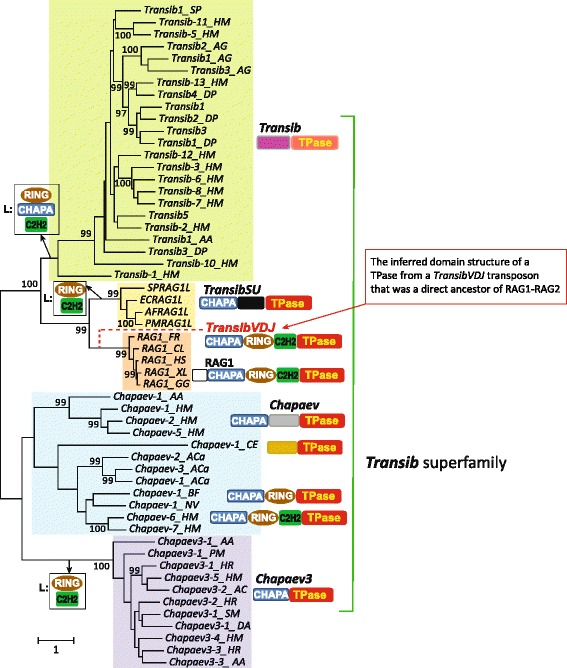
Phylogeny of Transib transposase core sequences. The Transib superfamily is composed of four clades: Transib, TransibSU, Chapaev and Chapaev3. The vertebrate RAG1 proteins cluster with the TransibSU clade. Species name abbreviations: HM - *Hydra magnipapillata*, NV - *Nematostella vectensis*, SP - *Strongylocentrotus purpuratus*, BF - *Branchiostoma floridae*, PM - *Petromyzon marinus*, CE - *Caenorhabditis elegans*, XL – *Xenopus laevis*, HS - *Homo sapiens*, FR - *Takifugu rubripes*, CL - *Carcharhinus leucas*, GG - *Gallus gallus*, AA - *Aedes aegypti*, AG - *Anopheles gambiae*, DP - *Drosophila pseudoobscura*, HR - *Helobdella robusta*, AC - *Anolis carolinensis*, ACa - *Aplysia californica*, Transib1-Transib5 are from *Drosophila melanogaster*. PMRAG1L is encoded by the Bat star *TransibSU-1_PM* transposon, SPRAG1L, ECRAG1L, AFRAG1L are TransibSU transposases from *Strongylocentrotus purpuratus*, *Evechinus chloroticus* sea urchin (encoded by the GenBank: GAPB01003278.1 transcribed RNA sequence) and *Asterias forbesi* starfish (GenBank: GAUS01036390.1 transcribed RNA sequence), respectively. In addition to Echinoderms, the TransibSU clade includes also a transposon from Pacific oyster (see Additional file 4; the oyster transposase core is ~40% identical to its homologues in Echinodermata). The tree was obtained using the PhyML: RtREV model, discrete gamma model with 10 categories and estimated gamma shape parameter, best of NNIs and SPRs tree topology search. Statistical support is indicated by aLRT-SH values above 95% [27]. Domain structure of RAG1 and transposases encoded by known members of the Transib superfamily was used to infer the domain structure of the transposase encoded by the TransibVDJ transposon, the hypothetical direct ancestor of the vertebrate RAG1-RAG2 locus. The losses of the CHAPA, RING and C2H2 domains in different clades that were inferred under the parsimony principle are indicated by “L:”.

Phylogenetic analysis of the Transib superfamily TPases produced a strongly supported clade that joined TransibSU (and the hypothetical TransibVDJ) with the vertebrate RAG1 genes (Figure 2). Thus, our findings reject the hypothesis that RAG2 was not a part of the “RAG-Transib transposon” [8,15] and are best compatible with the alternative hypothesis that both RAG1 and RAG2 were originally encoded within the same transposon [3,16,6,17,8,18].

*TransibVDJ* probably was recruited as the core of the V(D)J machinery some 500 MYA, at the base of the jawed vertebrate evolution. A second, much more recent recruitment of a *TransibSU* transposon most likely occurred in the purple sea urchin lineage (Figure 1E). Both SPRAG1L and SPRAG2L are expressed [13] but there is no no direct evidence that they are functional genes. The genomes of closely related species, including *Allocentrotus fragilis, Hemicentrotus pulcherrimus,* and *Strongylocentrotus franciscanus*, are not sequenced comprehensively. Therefore, it remains unclear whether or not these genomes contain orthologs of SPRAG1L and/or SPRAG2L. However, several short DNA sequences from each of these genomes that are available in GenBank (AG983548) and the NCBI Sequence Read Archive archive (SRA: SRR000294.264360, SRR000291.46691, SRR000328.260747, SRR000331.140516) are more than 90% identical to 200–300 fragments of the SPRAG1L and SPRAG2L CDSs (data not shown), indirectly supporting exaptation of a TransibSU transposon some 20 MYA (Figure 1E). Independent, relatively recent insertions of *TransibSUs*, followed by fossilization of the inserted elements, apparently occurred in the genomes of green sea urchin, starfish and oyster (and possibly also in many other species) (Figure 2). Taken together, our findings contradict and effectively refute the hypothesis of an ancient origin of the RAG1-RAG2 locus >650 MYA in the common ancestors of deuterostomes and chordates [13].

### Domain structure of the RAG1L protein encoded by the hypothetical direct transposon ancestor of the VDJ recombinase

The observations presented above clearly indicate that the RAG1-RAG2 locus evolved from a *Transib* superfamily transposon that encoded both proteins and was most closely related to the *TransibSU* transposons. However, the domain architecture of vertebrate RAG1 proteins differs from that of the RAG1 homologs encoded in *TransibSU*s (Figure 2). Specifically, RAG1 contains a RING finger domain that shows the ubiquitin ligase activity [19,20]. This domain is missing in *TransibSUs*. Thus, one might conjecture that RAG1 evolved via fusion between the TransibSU transposase and a RING finger derived from some other protein. However, comparison of the domain architectures of all known members of the *Transib* superfamily suggests a somewhat different evolutionary scenario (Figure 2). The canonical Transib transposase does not contain any identifiable domains other than the transposase core. The TransibSU transposase is so far the closest relative of the RAG1 core (>30% identity over ~600 aa). However, in addition to the absence of the RING finger, the TransibSU transposase contains only a truncated N-terminal part of RAG1 that is denoted Chapa domain after the *Chapaev* group of transposons [21] identified in the lancelet and starlet sea anemone genomes [8] (Figure 1E, Additional file 5). The Chapa domain represents a novel type of a highly complex zinc finger [21]. In two subgroups of *Chapaevs*, the Chapa domain is followed by a RING finger, similar to RAG1 (Figure 2). The Chapa domain is also present in *Chapaev3* transposons that are distantly related to the rest of the *Chapaevs* [22]. Importantly, RING finger and Chapa domain are not encoded in any other known transposable elements [23]. The transposases of the *Chapaevs* are only distantly related to the transposases of the other *Transibs*. Thus, comparison of the domain architectures of the Transib superfamily transposases (Figure 2) implies that the Chapa and RING-finger domains were already fused with the catalytic core in an ancient common ancestor of Chapaev, Transibs, TransibSUs and the hypothetical ancestor of the VDJ recombinases that can be denoted *TransibVDJ*. Apparently, the RING domain has been lost in all Transibs and *TransibSU*s, compatible with the existence of *TransibVDJ* transposons (Figure 2).

## Conclusions

The findings reported here strongly suggest that the direct ancestor of the vertebrate V(D)J recombinase was a hypothetical *TransibVDJ* transposon that encoded both the Transib transposase containing the RING domain (RAG1L protein) and the Kelch-PHD protein (RAG2L). The presence analysis strongly suggests that *TransibVDJ* was recruited as the core of the V(D)J machinery about 500 MYA, at the onset of the jawed vertebrate evolution. It is probably only a matter of time before members of the *TransibVDJ* group are identified in the genomes of some animals. While this manuscript was in preparation, a putative RAG1-RAG2-containing transposon has been reported as a polymorphic variant in a lancelet genome [24]. This putative element has not been described in any detail and so far is unavailable through public databases; subsequent analysis should elucidate its relationship with *TransibSU* or *TransibVDJ.*

The potential of the *Transib* transposons for genome rearrangement that is so efficiently exploited by the V(D)J recombination machinery is also the likely driving force behind their frequent fossilization and rare survival of active copies. Unless inactivated or put under tight control, these transposons can cause deleterious and potentially devastating genome instability.

## Methods

### Sequence analysis and phylogenetic tree construction

All sequences analyzed in our work are publicly available in GenBank. Protein sequences were aligned using MAFFT [25]. Local TBLASTN- and BLASTN-based sorted searches were performed using CENSOR [26]. Phylogenetic analysis was performed using PhyML with the RtREV model, a discrete gamma model with 10 categories and estimated gamma shape parameter, best of the NNIs and SPRs tree topology search; statistical support was approximated by aLRT-SH values [27]. The exon-intron structure of the RAG1L and RAG2L genes was predicted using FGENESH+ [28].

## Reviewers’ comments

### Reviewer 1: Mart Krupovic, Institut Pasteur, France

In this article, Kapitonov and Koonin report on the discovery of a new subgroup of Transib transposons (denoted TransibSU) that encode both RAG1- and RAG2-like proteins. The authors present compelling evidence for the mobility of these transposons and narrow down on the organization of the transposon that gave rise to the V(D)J recombination machinery of jawed vertebrates. This is an important discovery which allows putting to rest the hypothesis that RAG2 gene was not part of the ‘RAG transposon’ (1) and instead supports the alternative possibility that the ‘RAG transposon’ contained genes for both RAG1 and RAG2 (2,3). The article is very well written and I have only a few minor comments:

The Methods section is missing.

Authors’ response: We added a brief Methods section.

Figure 2: how was the species tree obtained? The definition of the magenta ellipse is not provided.

Authors’ response: The tree topology in Figure *1**E follows the commonly accepted species phylogeny of sea urchins and other deuterostomes that is maintained by the sea urchin genome database* [14] *and EchinoBase (**http://www.echinobase.org/Echinobase/**). We added the definition of the magenta ellipse in Figure**1**E: it corresponds to the uncharacterized RAG1-RAG2-transposon identified recently as a polymorphic insertion in a lancelet genome* [24].

Pointers to Figure 1E on lines 120, 125 and 132 should be substituted with Figure 2.

Authors’ response: *pointers corrected*.

Line 34: “b-propeller” should be “beta-propeller”.

Authors’ response: *Done*.

References:

1. Fugmann SD. The origins of the Rag genes — from transposition to V(D)J recombination. Semin Immunol. 2010; 22:10–16.

2. Schatz DG. Antigen receptor genes and the evolution of a recombinase. Semin Immunol. 2004; 16(4):245–56.

3. Koonin EV, Krupovic M. Evolution of adaptive immunity from transposable elements combined with innate immune systems. Nat Rev Genet. 2015; 16(3):184–92.

Authors’ response: *We added these three references*.

### Reviewer 2: I. King Jordan, Georgia Institute of Technology, United States of America

Kapitonov and Koonin report on the discovery of single transposons that encode homologs of both the RAG1 and RAG2 subunits of V(D)J recombinase. These RAG1-RAG2 encoding transposons are members of the Transib superfamily of elements and were uncovered in three different species. The evolutionary origin of RAG1 from a Transib element was previously established, but a transposon encoding both RAG1 and RAG2 had never been found before. This is an important and noteworthy discovery as it further solidifies the hypothesis that the emergence of the adaptive immune system in vertebrates was based on the co-option of proteins that were once encoded by ‘selfish’ genetic elements.

The conclusions drawn in the manuscript, which appear to be quite solid overall, rest on an impressively detailed analysis of recently sequenced genomes that are apparently incompletely assembled and annotated. While the report is succinct, the authors do provide substantial supporting evidence for their interpretations of the data in the Additional files.

The authors briefly mention an alternative hypothesis for the ancient origin of the RAG1-RAG2 gene locus, which was published subsequent to the original report of the transposon origins of RAG1. This scenario is implicitly rejected by their work, but it may be helpful to have a more explicit articulation of how these two interpretations of the *S. purpuratus* findings differ.

Authors’ response: *To emphasize the differences between these two scenarios we included an explicit discussion of this point.*

The extent to which Transib transposons appear to be inactivated and rapidly fossilized is curious and raises a couple of issues with the sequence analysis and interpretation of the results. The authors mention that “since autonomous Transib transposons are typically present in animal genomes in only one or at most a few copies”, it is not surprising that the element termini are not detectable. This may indeed be the case, but shouldn’t they therefore expect to find at least one autonomous copy of a RAG1-RAG2 element in the genomes that they analyzed?

Authors’ response: *In the vast majority of sequenced animal genomes, autonomous Transibs are not present at all. In the genomes that do contain autonomous Transibs, these transposons are usually represented by the small numbers of copies, often damaged by mutations. This is likely to indicate that transposition of Transibs is tightly regulated by the host. There is no expectation to find at least one autonomous copy of a RAG1-RAG2 element in the analyzed genomes. In many animal populations, active autonomous transposons are probably not fixed. Also, the turnover of transposons is extremely high so that autonomous transposons that were transpositionally active several million years ago are likely to have been be lost.*

The rationale behind the concluding statement that “the potential of the Transib transposons for genome rearrangement that is so efficiently exploited by the V(D)J recombination machinery is also the driving force behind their frequent fossilization and rare survival of active copies” is not entirely clear. My understanding is that this catalytic activity serves to transpose the Transib elements and would have been co-opted later to perform V(D)J recombination based on interaction with terminal inverted repeat-derived recombination signal sequences. Thus, efficient activity of RAG1-RAG2 in Transib transposons may actually be expected to lead to accumulation of multiple autonomous and non-autonomous copies, as seen with autonomous transposons and derivative MITEs in plants for example, particularly for the kinds of relatively large metazoan genomes analyzed here. Is there any evidence to suggest that transposon encoded RAG1-RAG2 complexes could catalyze additional genome re-arrangements (i.e. beyond transposition)?

Authors’ response: *As mentioned above, the low activity of Transibs in most metazoans is likely to indicate tight regulation by the host. Therefore, “efficient activity of RAG1-RAG2 in Transibs” actually can be expected to lead to suppression of transposition. We are unaware of direct evidence of the ability of the transposon-encoded RAG1-RAG2 proteins to promote additional genome rearrangements, beyond transposition. Some indirect evidence seems to exist, though. For example, in the starfish and oyster genomes, the genes encoding RAG1L transposase and RAG2L protein, respectively, are partially duplicated and inverted.*

It is not clear from Figure 1B as to whether Scaffold71 of L. variegates also encodes the amino termini of LVRAG1L and LVRAG2L. How is it that the two scaffolds in the figure were not originally assembled? Do they really overlap to the extent that is shown in the figure?

Authors’ response: *Scaffold71 encodes the N-terminus of LVRAG2L (aa pos. 1–184) only at nucleotide positions 91264–90188. It does not overlap Scaffold19893. The 102-bp terminus of Scaffold19893 (pos. 102–1) is the 3’-terminus of an intron, whose complete sequence is not known. The 5’-terminus of this intron is the position 90187 of Scaffold71.*

## Additional files

Additional file 1:
**LRRAG1L and LVRAG2L genes in the Green Sea urchin** ***Lytechinus variegatus*** **genome.** A), Leftovers of the LVRAG1 gene identified in Scaffold19893 code for the RAG1/Transib transposase core ~55% identical to SPRAG1L (pos. 420–978). The LVRAG1L core is damaged by 4 premature stop codons and two frameshifts. B), Prediction of the exon-intron structure of LVRAG2L by FGENESH+ [28]. The complete LVRAG2L gene is predicted in a sequence named combo2 that was assembled from Scaffold19873 and Scaffold71. C), BLASTP similarity between LVRAG2L and SPRAG2L proteins. D), DNA sequence of combo2. Additional file 2:
**PMRAG1L and PMRAG2L genes in the the Bat star** ***Patiria miniata*** **genome.** A), Exon-intron structure and protein sequence of PMRAG1L predicted by FGENESH+ in the assembly of the AKZP01053170 - AKZP01053172 contigs fused together. B) Similarity between PMRAG1L and SPRAG1L proteins. C) Exon-intron structure and protein sequence of PMRAG2L predicted in the AKZP01064647 contig. D) Similarity between PMRAG2L and SPRAG2L proteins. Additional file 3:
**TIRs identified in the Bat star** ***TransibSU-1_PM*** **transposon.** A) Pairwise alignment of the transposon termini taken in different orientation. The scaffold GenBank accessions and nucleotide positions are listed in the first column. B) The map of the *TransibSU-1_PM* copies identified by Censor [26] in the Bat star genome. First three columns – genome coordinates of the identified copies. Column four – name of the 12401-bp query sequence. Columns 5 and 6 – positions of the query region similar to the particular copy. Column 7 – orientation of the copy. Column 8 – DNA identity between the copy and the query sequence. Additional file 4:
**Remnants of the TransibSU transposon identified in the Pacific oyster** ***Crassostrea gigas*** **genome.** A) Map of RAG1L- and RAG2L-coding regions (TBLASTN /Censor) is combined with the map of known oyster transposable elements [23]. B) Similarity of the oyster RAG1L and RAG2L proteins to SPRAG1L and SPRAG2L. C) Recent duplication and inversion of the RAG1L-encoding region. Additional file 5:
**Multiple alignment of N-terminal parts of the Transib superfamily transposases and RAG1s.**

## Abbreviations

RAG1: Recombination activating gene 1
RAG2: Recombination activation gene 2
RAG1L: RAG1-like
RAG2L: RAG2-like
SPRAG1L and SPRAG2L: RAG1L and RAG2L proteins encoded by the transposon in the Strongylocentrotus purpuratus genome
LVRAG1L and LVRAG2L: RAG1L and RAG2L proteins encoded by the transposon in the Lytechinus variegatus genome
PMRAG1L and PMRAG2L: RAG1L and RAG2L proteins encoded by a transposon in the Patiria minata genome
TIR: Terminal inverted repeat
RSS: Recombination signal sequence
MYA: Million years ago
