# Supplementary material for: Evolution of the RAG1-RAG2 locus: both proteins came from the same transposon
Source: Biol Direct. 2015 Apr 28;10:20. doi: 10.1186/s13062-015-0055-8 (PMC4411706; doi:10.1186/s13062-015-0055-8)
Supplement: Additional file 5: — Multiple alignment of N-terminal parts of the Transib superfamily transposases and RAG1s. [file 13062_2015_55_MOESM5_ESM.pdf]

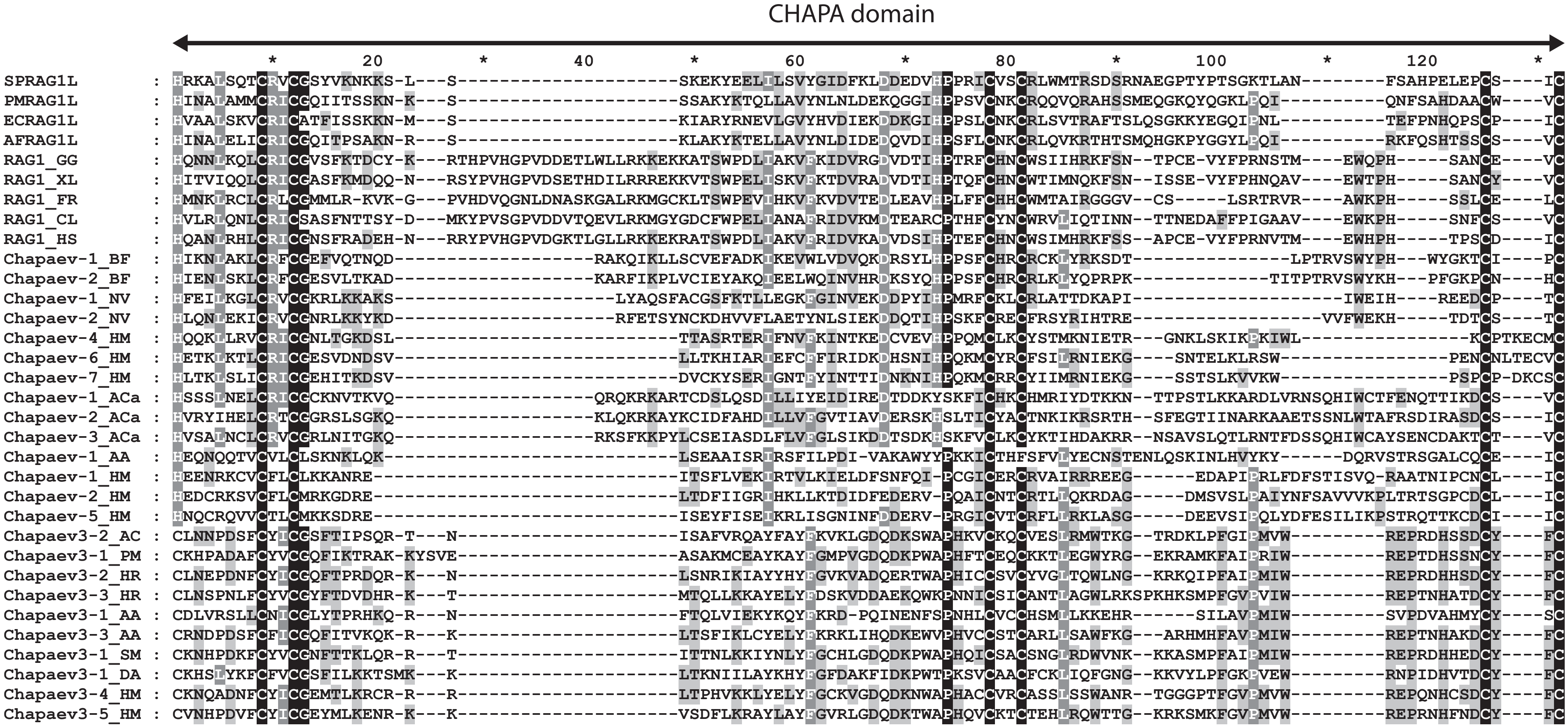
CHAPA domain
* 20 * 40 * 60 * 80 * 100 * 120 *
SPRAG1L : HRKALSQTCRVCGSYVKNKKS-L---S--------------------SKEKYEELILSVYGIDFKLDDEDVHEPRICVSCRLWMTRSDSRNAEGPTYPTSGKTLAN--------FSAHPELEPCS----IC
PMRAG1L : HINALAMMCRICGQIITSSKN-K---S--------------------SSAKYKTQLLAVYNLNLDEKQGGIHPPSVCNKCRQQVQRAHSSMEQGKQYQGKLPQI---------QNFSAHDAACW----VC
ECRAG1L : HVAALSKVCRICATFISSKKN-M---S--------------------KIARYRNEVLGVYHVDIEKDDKGIHPPSLCNKCRLSVTRAFTSLQSGKKYEGQIPNL---------TEFPNHQPSCP----IC
AFRAG1L : HINALELICRICGQITPSAKN-R---S--------------------KLAKYKTELLAVYNLDIDEDQVDIHPSFLCNKCRLQVKRTHTSMQHGKPYGGYLPQI---------RKFQSHTSSCS----VC
RAG1_GG : HQNNLKQLCRICGVSFKTDCY-K---RTHPVHGPVDDETLWLLRKKEKKATSWPDLIAKVFKIDVRGDVDTIHPTRFCHNCWSIIHRKFSN--TPCE-VYFPRNSTM----EWQPH-----SANCE----VC
RAG1_XL : HITVIQQLCRICGASFKMDQQ-N---RSYPVHGPVDSETHDILRRREKKVTSWPELISKVFKTDVRADVDTIHPTQFCHNCWTIMNQKFSN--ISSE-VYFPHNQAV----EWTPH-----SANCY----VC
RAG1_FR : HMNKLRCLCRLCGMMLR-KVK-G---PVHDVQGNLDNASKGALRKMGCKLTSWPEVIHKVFKVDVTEDLEAVHPLFFCHHCWMTAIRGGGV--CS-----LSRTRVR----AWKPH-----SSLCE----LC
RAG1_CL : HVLRLQNLCRICSASFNTTSY-D---MKYPVSGPVDDVTQEVLRKMGYGDCFWPELIANAFRIDVKMDTEARCPTHFCYNCWRVLIQTINN--TTNEDAFFPIGAAV----EWKPH-----SNFCS----VC
RAG1_HS : HQANLRHLCRICGNSFRADEH-N---RRYPVHGPVDGKTLGLLRKKEKRATSWPDLIAKVFRIDVKADVDSIHPTEFCHNCWSIMHRKFSS--APCE-VYFPRNVTM----EWHPH-----TPSCD----IC
Chapaev-1_BF : HIKNLAKLCRFCGEFVQTNQD------------------RAKQIKLLSCVEFADKIKEVWLVDVQKDRSYLHPPSFCHRCRCKLYRKSDT---------------LPTRVSWYPH---WYGKTCI----PC
Chapaev-2_BF : HIENLSKLCRFCGESVLTKAD------------------KARFIKPLVCIEYAKQIEELWQINVHRDKSYQHPPSFCHRCRLKLYQPRPK-------------TITPTRVSWYKH---PFGKPCN----HC
Chapaev-1_NV : HFEILKGLCRVCGKRLKKAKS--------------------LYAQSFACGSFKTLLEGKFGINVEKDDPYIHPMRFCKLCRLATTDKAPI--------------------IWEIH----REEDCP----TC
Chapaev-2_NV : HLQNLEKICRVCGNRLKKYKD--------------------RFETSYNCKDHVVFLAETYNLSIEKDDQTIHPSKFCRECFRSYRIHTRE------------------VVFWEKH-----TDTCS----TC
Chapaev-4_HM : HQQKLLRVCRICGNLTGKDSL--------------------------TTASRTERIFNVFKINTKEDCVEVHPPQMCLKCYSTMKNIETR---GNKLSKIKPKIWL---------------KCPTKECMC
Chapaev-6_HM : HETKLKTLCRICGESVDNDSV--------------------------LLTKHIARIEFCFFIRIDKDHSNIHPQKMCYRCFSILRNIEKG----SNTELKLRSW---------------PENCNLTECVC
Chapaev-7_HM : HLTKLSLICRICGEHITKDSV--------------------------DVCKYSERIGNTFYINTTIDNKNIHPQKMCRRCYIIMRNIEKG----SSTSLKVVKW---------------PSPCP-DKCSC
Chapaev-1_ACa : HSSSLNELCRICGCKNVTKVQ------------------QRQKRKARTCDSLQSDILLIYEIDIREDTDDKYSKFICHKCHMRIYDTKKN--TTPSTLKKARDLVRNSQHIWCTFENQTTIKDCS----VC
Chapaev-2_ACa : HVRYIHELCRTCGGRSLSGKQ------------------KLQKRKAYKCIDFAHDILLVFGVTIAVDERSKHSLTICYACTNKIKRSRTH--SFEGTIINARKAAETSSNLWTAFRSDIRASDCS----IC
Chapaev-3_ACa : HVSALNCLCRVCGRLNITGKQ------------------RKSFKKPYLCSEIASDLFLVFGLSIKDDTSDKHSKFVCLKCYKTIHDAKRR--NSAVSLQTLRNTFDSSQHIWCAYSENCDAKTCT----VC
Chapaev-1_AA : HEQNQQTVCVLCLSKNKLQK--------------------------LSEAAISRIRSFILPDI-VAKAWYYPKKICTHFSFVLYECNSTENLQSKINLHVYKY------DQRVSTRSGALCQCE----IC
Chapaev-1_HM : HEENRKCVCFLCLKKANRE---------------------------ITSFLVEKIRTVLKIELDFSNFQI-PCGICERCRVAIRRREEG------EDAPIPRLFDFSTISVQ-RAATNIPCNCL----IC
Chapaev-2_HM : HEDCRKSVCFLCMRKGDRE---------------------------LTDFIIGRIHKLLKTDIDFEDERV-PQAICNTCRTLIQKRDAG-----DMSVSLPAIYNFSAVVVKPLTRTSGPCDCL----IC
Chapaev-5_HM : HNQCRQVVCTLCMKKSDRE---------------------------ISEYFISEIKRLISGNINFDDERV-PRGICVTCRFLIRKLASG-----DEEVSIPQLYDFESILIKPSTRQTTKCDCI----IC
Chapaev3-2_AC : CLNNPDSFCYICGSFTIPSQR-T---N--------------------ISAFVRQAYFAYFKVKLGDQDKSWAPHKVCKQCVESLRMWTKG--TRDKLPFGIPMVW---------REPRDHSSDCY----FC
Chapaev3-1_PM : CKHPADAFCYVCGQFIKTRAK-KYSVE--------------------ASAKMCEAYKAYFGMPVGDQDKPWAPHFTCEQCKKTLEGWYRG--EKRAMKFAIPRIW---------REPTDHSSNCY----FC
Chapaev3-2_HR : CLNEPDNFCYICGQFTPRDQR-K---N--------------------LSNRIKIAYYHYFGVKVADQERTWAPHICCSVCYVGLTQWLNG--KRKQIPFAIPMIW---------REPRDHHSDCY----FC
Chapaev3-3_HR : CLNSPNLFCYVCGYFTDVDHR-K---T--------------------MTQLLKKAYELYFDSKVDDAEKQWKPNNICSICANTLAGWLRKSPKHKSMPFGVPVIW---------REPTNHATDCY----FC
Chapaev3-1_AA : CDLVRSLLCNICGLYTPRHKQ-R---N--------------------FTQLVIEKYKQYFKRD-PQINENFSPNHLCVCCHSMILKKEHR------SILAVPMIW---------SVPDVAHMYCY----SC
Chapaev3-3_AA : CRNDPDSFCFICGQFITVKQK-R---K--------------------LTSFIKLCYELYFKRKLIHQDKEWVPHVCCSTCARLLSAWFKG---ARHMHFAVPMIW---------REPTNHAKDCY----FC
Chapaev3-1_SM : CKNHPDKFCYVCGNFTTKLQR-R---T--------------------ITTNLKKIYNLYFGCHLGDQDKPWAPHQICSACSNGLRDWVNK--KKASMPFAIPMIW---------REPRDHHEDCY----FC
Chapaev3-1_DA : CKHSLYKFCFVCGSFILKKTSMK---K--------------------LTKNIILAYKHYFGFDAKFIDKPWTPKSVCAACFCKLIQFGNG--KKVYLPFGKPVEW---------RNPIDHVTDCY----FC
Chapaev3-4_HM : CKNQADNFCYICGEMTLKRCR-R---R--------------------LTPHVKKLYELYFGCKVGDQDKNWAPHACCVRCASSLSSWANR--TGGGPTFGVPMVW---------REPQNHCSDCY----FC
Chapaev3-5_HM : CVNHPDVFCYICGEYMLKENR-K---K--------------------VSDFLKRAYLAYFGVRLGDQDKTWAPHQVCKTCTEHLRQWTTG--KRKSMKFGVPMVW---------REPRNHFNDCY----FC

```
                     140        *        160        *        180        *        200        *        220        *        240        *        260
SPRAG1L       : EAYQATKS--TKRKA-VGTDG--------------LPPPKIPSAAVSGT-------------------------------------------------------------------DEQQASCSFTAPSPPT
PMRAG1L       : SAYTAHV---AQLRP-QFAAH-----------------QSSSSDQPDQP-----------------------------------------------------------------------------------
ECRAG1L       : LEHRVRL---NSIQP-VFKKP--------------AALPSTLTSGSSQR-----------------------------------------------------------------------------------
AFRAG1L       : RTHATHK---SLLQP-QFAAAGPSTPTSAAPPELQAGPSSSTTSAPPEL-----------------------------------------------------------------------------------
RAG1_GG       : HTPS------RGVKR-KSQ------------------PPNVQHGKRVKI------------IAERARVNRG--IKNQV---IKNKNV--------MKEITNCKNRHLSTKLL-----------AVDYPADFI
RAG1_XL       : HSSK------PWGKR-KSAPQ---------------LNPHKMKKRKRGPE------------FVKKSKTSSGNSIQWKN---MKAFNQ--------MKD--SCKKIHLDNNLL-----------VLDYPSDFV
RAG1_FR       : SPRKVSFERTRRKRR-KGV------------------PRALSLGKRTRCDNSDGNADGERRNLERP-------ALRDW---PKSRDQREQW----VRSITHCQRDHLSINLI-----------SEKLPEDFL
RAG1_CL       : NFSS------GTRKR-KGL------------------KRSPPAAKKAKV------------TVETP-------IRPGV---IKRKDQKLSTSIMLAKKIVNCKKIHLTSKLL-----------VLDYPNNFI
RAG1_HS       : NTAR------RGLKR-KSL------------------QPNLQLSKKLKT------------VLDQARQARQ--RKRRAQARISSKDV--------MKKIANCSKIHLSTKLL-----------AVDFPEHFV
Chapaev-1_BF  : EKVATLT---KGGRP-VKKKS--------------PGRPKTQKNEKGKN-------------------------------------EKGEKVEECAKTMSEETFSELLSTLDDFPLDEDRFK-----PICSL
Chapaev-2_BF  : EKVLSISK--GGKKP-KKKSK--------------AGRPRLQTNDKGAK-----------------------------------VEQEQSTPEYSDGNLTEETFAHLLDLLEDLPFDETKFE-----PICNL
Chapaev-1_NV  : ALEEQIQ---KGGRP-KKAKR--------------GGARGQAKNSAWEE-------------------------------------------TTNAGLKAQLIARVTELSASMNTLRDHRFTERFDFFETKK
Chapaev-2_NV  : EKEEFAR---KGGRP-KKSKR--------------GGGNTRKAQRRISF----------------------------------------------VDSVAALQDQIADVTKEIASRRIDNFSERFELNEVRK
Chapaev-4_HM  : IYG-------KVGRK-PSPN---------------VGRPGVF------------------------------------------------------KRWTQLNINLFLESLPLPL-NKKEISEFNIQLNPHI
Chapaev-6_HM  : FKS-------NKGRK-KKKKI--------------SGRPSSISYKE--------------------------------------------------NIWTRHKINNIQSKLSPQTKLNLKLQDINCILNPHV
Chapaev-7_HM  : FSK-------VSGRK-VKKCK--------------AGRPCIIEQNQ--------------------------------------------------KRWTRPIINNLIASIPKQT-LRLNAIDIDPVANPHL
Chapaev-1_ACa : RHRVNLS---DGCLK-PAKEP--------------AATVAGDSPSTSPS---------------------------------LHSQHSPVTPSQTTSTYDDPTTR---------------------------
Chapaev-2_ACa : THYHRTS---MGSKN-TPIEL--------------VTTKTSEATKTTHS---------------------------------PHRSTFEDSANTTSNSTAEPSTH---------------------------
Chapaev-3_ACa : EQKRLLS---LGCAT-VKPRD--------------PQSHSSSHEKYTHQ----------------------------------------ASPSTNISETDTTTQSTCTRALSTDSTQQNDPH----------
Chapaev-1_AA  : IAARVTLP--NLIDPQKKSRK--------------RGRPAVLEESKRAR-----------------------------------------------------------------------------------
Chapaev-1_HM  : QTARTNM---NQRHP--------------------LEPPKPKENS---------------------------------------------------------------------------------------
Chapaev-2_HM  : QIAKLKL---NQKHP--------------------LA-PKEQTRNLDQG-----------------------------------------------------------------------------------
Chapaev-5_HM  : QISKTKG---KGKHP--------------------FEKPSQQEVQKEEK-----------------------------------------------------------------------------------
Chapaev3-2_AC : IVKT------SGYNK-KNKCK--------------IEYPSLPSAIRPVP---HSA-EIPVPVFIE-------------------------------------------------------------------
Chapaev3-1_PM : MVDP------SKRRTGKNAPA--------------ITYPDLPSSIAPVP---HCH-ELPVPTPPE-------------------------------------------------------------------
Chapaev3-2_HR : MTNI------QGHSK-KTKAS--------------IVYPNCSSAIKPVP---HSV-EYPVPTPPT-------------------------------------------------------------------
Chapaev3-3_HR : MTVI------KGFSF-KTRKS--------------ISYPDIESVSKPIP---HNPVNCPVPISPD-------------------------------------------------------------------
Chapaev3-1_AA : LMPSL-----VGRKW-CQRSE--------------IQYPKRSLCTLPEKVLP--------------------------------------------------------------------------------
Chapaev3-3_AA : LVTV------QGIST-KNKKM--------------ICYPNVRSVDQPTP---HSN-DLPIPVPPN-------------------------------------------------------------------
Chapaev3-1_SM : SVNM------TGFST-KNKNK--------------IVYPVMDSARRPVQ---HGE-ELPIPIPPD-------------------------------------------------------------------
Chapaev3-1_DA : LTKV------QG----GKHFK--------------VIYPDVQSVTKAAP---HSS-LYPKREPPA-------------------------------------------------------------------
Chapaev3-4_HM : SVNI------SGRNS-KGKKA--------------IVYPNLPSAIRPVP---HSA-ELTVPEPPS-------------------------------------------------------------------
Chapaev3-5_HM : ILNI------TGINR-NNRSK--------------WTYPDLLSARRPVL---HSE-EVPIPTFLQ-------------------------------------------------------------------
```

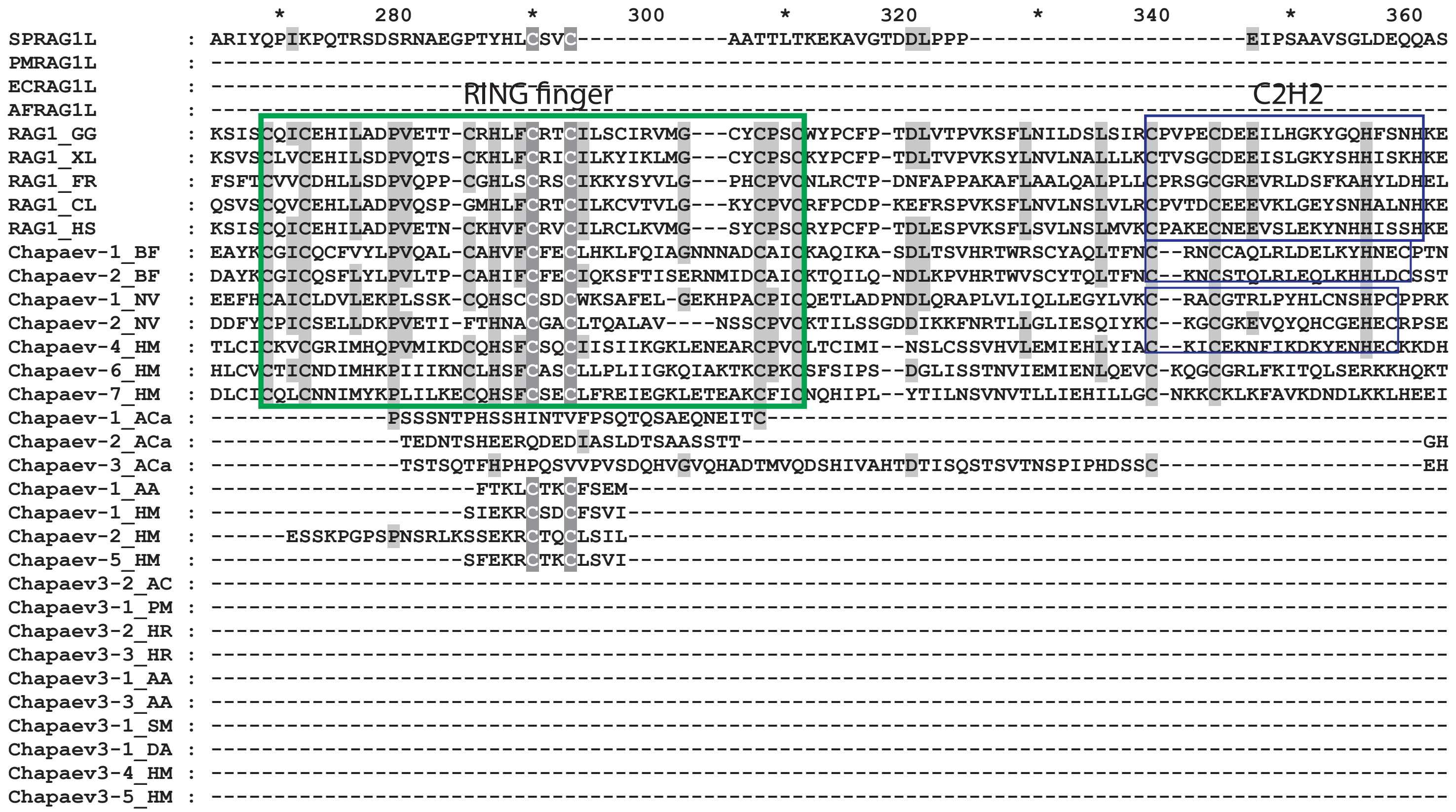

RING finger
C2H2
* 280 * 300 * 320 * 340 * 360
SPRAG1L : ARIYQPIKPQTRSDSRNAEGPTYHLCSVC------------AATTLTKEKAVGTDDLPPP----------------------EIPSAAVSGLDEQQAS
PMRAG1L : ---
ECRAG1L : ---
AFRAG1L : ---
RAG1_GG : KSISCQICEHILADPVETT-CRHLFCRTCILSCIRVMG---CYCPSCWYPCFP-TDLVTPVKSFLNILDSLSIRCPVPECDEEILHGKYGQHFSNHKE
RAG1_XL : KSVSCLVCEHILSDPVQTS-CKHLFCRICILKYIKLMG---CYCPSCKYPCFP-TDLTVPVKSYLNVLNALLLKCTVSGCDEEISLGKYSHHISKHKE
RAG1_FR : FSFTCVVCDHLLSDPVQPP-CGHLSCRSCIKKYSYVLG---PHCPVCNLRCTP-DNFAPPAKAFLAALQALPLLCPRSGCGREVRLDSFKAHYLDHEL
RAG1_CL : QSVSCQVCEHLLADPVQSP-GMHLFCRTCILKCVTVLG---KYCPVCRFPCDP-KEFRSPVKSFLNVLNSLVLRCPVTDCEEEVKLGEYSNHALNHKE
RAG1_HS : KSISCQICEHILADPVETN-CKHVFCRVCILRCLKVMG---SYCPSCRYPCFP-TDLESPVKSFLSVLNSLMVKCPAKECNEEVSLEKYNHHISSHKE
Chapaev-1_BF : EAYKCGICQCFVYLPVQAL-CAHVFCFECLHKLFQIAGNNNADCAICKAQIKA-SDLTSVHRTWRSCYAQLTFNC--RNCCAQLRLDELKYHNECPTN
Chapaev-2_BF : DAYKCGICQSFLYLPVLTP-CAHIFCFECIQKSFTISERNMIDCAICKTQILQ-NDLKPVHRTWVSCYTQLTFNC--KNCSTQLRLEQLKHHLDCSST
Chapaev-1_NV : EEFHCAICLDVLEKPLSSK-CQHSCCSDCWKSAFEL-GEKHPACPICQETLADPNDLQRAPLVLIQLLEGYLVKC--RACGTRLPYHLCNSHPCPPRK
Chapaev-2_NV : DDFYCPICSELLDKPVETI-FTHNACGACLTQALAV----NSSCPVCKTILSSGDDIKKFNRTLLGLIESQIYKC--KGCGKEVQYQHCGEHECRPSE
Chapaev-4_HM : TLCICKVCGRIMHQPVMIKDCQHSFCSQCIISIIKGKLENEARCPVCLTCIMI--NSLCSSVHVLEMIEHLYIAC--KICEKNFIKDKYENHECKKDH
Chapaev-6_HM : HLCVCTICNDIMHKPIIIKNCLHSFCASCLLPLIIGKQIAKTKCPKCSFSIPS--DGLISSTNVIEMIENLQEVC-KQGCGRLFKITQLSERKKHQKT
Chapaev-7_HM : DLCICQLCNNIMYKPLILKECQHSFCSECLFREIEGKLETEAKCFICNQHIPL--YTILNSVNVTLLIEHILLGC-NKKCKLKFAVKDNDLKKLHEEI
Chapaev-1_ACa : --------------PSSSNTPHSSHINTVFPSQTQSAEQNEITC---
Chapaev-2_ACa : ---------------TEDNTSHEERQDEDIASLDTSAASSTT---GH
Chapaev-3_ACa : ---------------TSTSQTFHPHPQSVVPVSDQHVGVQHADTMVQDSHIVAHTDTISQSTSVTNSPIPHDSSC---------------------EH
Chapaev-1_AA : ---------------------FTKLCTKCFSEM--
Chapaev-1_HM : --------------------SIEKRCSDCFSVI--
Chapaev-2_HM : ------ESSKPGPSPNSRLKSSEKRCTQCLSIL--
Chapaev-5_HM : --------------------SFEKRCTKCLSVI--
Chapaev3-2_AC : ---
Chapaev3-1_PM : ---
Chapaev3-2_HR : ---
Chapaev3-3_HR : ---
Chapaev3-1_AA : ---
Chapaev3-3_AA : ---
Chapaev3-1_SM : ---
Chapaev3-1_DA : ---
Chapaev3-4_HM : ---
Chapaev3-5_HM : ---
